# Dynamic Changes in Proteome during Yak Oocyte Maturation Analyzed Using iTRAQ Technology

**DOI:** 10.3390/ani13132085

**Published:** 2023-06-23

**Authors:** Xin Ma, Meng Wang, Jinglei Wang, Qian Zhang, Sisi Pu, Rui Wang, Sijiu Yu, Libin Wang, Yangyang Pan

**Affiliations:** 1 College of Veterinary Medicine, Gansu Agricultural University, Lanzhou 730070, China; mxinmaxin@163.com (X.M.); wangmeng@gsau.edu.cn (M.W.); w231012gsau@163.cn (J.W.); zq880204@126.com (Q.Z.); pssummer0602@163.com (S.P.); 17693109040@163.com (R.W.); sijiuy@126.com (S.Y.); wanglb@gsau.edu.cn (L.W.); 2 Gansu Province Livestock Embryo Engineering Research Center, Lanzhou 730070, China

**Keywords:** oocytes, iTRAQ, yak, differential proteomics

## Abstract

**Simple Summary:**

The regulation of proteins at different time points during oocyte maturation varies. In this study, dynamic changes in oocytes at the GV, MI, and MII stages during oocyte maturation were analyzed using iTRAQ technology. Protein functions from the GV stage to the MI stage were mainly concentrated in the metabolic pathway, and protein functions from the MI stage to the MII stage were mainly concentrated in the regulation of meiosis and genetic material preparation. The results showed that there were transient dynamic changes in the proteome during yak oocyte maturation, and that the physiological functions mediated by these changes were also different. The accurate identification of the differential proteins in the three stages of GV, MI, and MII provided basic information for studying protein regulation at each time point during the maturation of yak oocytes and the molecular mechanism of in vitro maturation.

**Abstract:**

The aim of this study was to investigate protein regulation at different time points during the in vitro maturation of yak oocytes. Yak oocytes at GV, MI, and MII stages were collected during in vitro maturation, and differential proteomics sequencing was performed using iTRAQ technology. GO functional classification indicated that the differential proteins were closely associated with biological processes such as “metabolic processes”, and molecular events such as “binding” molecular-function-related categories were active. KOG analysis showed that energy-metabolism-related activities were vigorous during oocyte development from the GV phase to MI phase, and genetic material preparation activities were more active when oocytes developed from the MI stage to MII stage. KEGG pathway analysis showed that the PPAR metabolic pathway, Hippo signaling pathway, and ECM–receptor interaction and metabolic pathway were enriched from the GV to the MI stages. The PI3K-Akt, TGF-β, and phagosome pathways were enriched from the MI stage to the MII stage. These results indicate that transient dynamic changes occurred in the proteome during the maturation of yak oocytes, and the physiological functions mediated by these were also different. The accurate identification of the differential proteins in the three stages of GV, MI, and MII was helpful in further analyzing the molecular regulatory mechanism of yak oocyte maturation.

## 1. Introduction

Yak is a unique species distributed in the alpine region of the Qinghai–Tibet Plateau in China. At present, 95% of all yaks live in Qinghai, Tibet, Gansu, and other provinces in China, and it is the only cattle animal adapted to high-altitude areas [1]. Yaks provide necessary meat, milk, fur, and other production and living materials for herders on the Qinghai–Tibet Plateau. However, owing to the harsh environment of intense cold and low oxygen and the relatively primitivity of the species, the natural fertility of yaks is low, with an average of only 48%. More than one half of yaks give birth to one calf every two years or two calves every three years [2,3]. Therefore, the application of modern reproductive technologies, such as embryo transfer, in vitro fertilization, and somatic cell nuclear transfer, will bring qualitative changes to the rapid propagation of yak to increase their economic value [4,5,6]. Oocytes can not only provide genetic material for mammalian reproduction, but also provide the necessary nutrients for early embryonic development. Their maturity can have a direct impact on the normal development of early embryos. The in vitro maturation of oocytes is a basic link to modern breeding technology. The quality of oocytes after in vitro maturation directly affects the subsequent fertilization process and development of early embryos [7,8]. At present, compared with plain cattle, the cleavage rate and blastocyst rate of yak oocytes obtained via in vitro maturation are lower after in vitro fertilization, which seriously hinders the promotion and application of embryo transfer technology in yak. Oocytes are important female germ cells, and many protein changes are involved in in vitro maturation. Therefore, it is crucial to study the dynamic changes in proteins during each period of oocyte maturation to improve yak fertility [9,10,11]. iTRAQ is a protein quantification technology based on tandem mass spectrometry that can identify almost all proteins in an animal [12,13]. iTRAQ quantitative differential proteomics is currently widely used, as it can identify the differential proteins in cells at different stages and provides a new method for further investigating life phenomena [14]. The differential proteome of mammalian oocytes has been studied in humans, mice, sheep, and buffalo [15,16,17]. Through iTRAQ technology, we can screen out proteins with significant differences in expression during oocyte in vitro maturation, and understand their functions during oocyte maturation, which is helpful for a more detailed analysis of molecular mechanisms. Although there have been simple reports on the proteomics of buffalo, only some differential proteins have been obtained and not too many specific pathways have been involved [18]. However, research on yak oocytes has mostly focused on a single gene protein, and research on differential proteomics has rarely been reported. The differential proteomics of mammalian oocytes is mainly focused on the comparison of the GV and MII stages and rarely involves the MI stage; this approach misses the dynamic changes in some proteins during oocyte maturation [19,20]. Therefore, in the present study, iTRAQ technology was used to study the differential proteomics of yak oocytes at the GV, MI, and MII stages. It helped improve the proteome database of mammalian oocytes and provided a basis for the further discovery of the molecular regulatory mechanism of yak oocyte development and maturation.

## 2. Materials and Methods

### 2.1. Collection and In Vitro Culture of Oocytes

The experimental samples were collected from a slaughterhouse in Xining. After the yaks were slaughtered, the ovaries were immediately collected, rinsed, and stored in saline with penicillin and streptomycin. The yak ovaries were transported to the laboratory stored in an incubator with warm water at 37 °C for two hours. After washing the ovaries thrice with normal saline, follicles with a diameter of 3–10 mm on the surface of the ovaries were extracted using an 18 G needle syringe. The cumulus–oocyte complexes (COCs) with intact morphology, uniform cytoplasm, and three layers of dense cumulus cells were selected under a stereomicroscope and washed three times with washing solution (DPBS + 5% FBS). Then, they were transferred to a well-balanced maturation medium (M199 1 μg/mL LH + 1 μg/mL FSH + 1 μg/mL E2 + 1 μg/mL EGF + 0.2 μg/mL sodium pyruvate + 0.03 μg/mL NaOH + 0.03 μg/mL glucose + 10% FBS). The COCs at GV (0 h), MI (12 h), and MII (24 h) stages were collected, and the granulosa cells were removed using 0.1% hyaluronidase and placed in RNase-free tubes [21].

### 2.2. Experimental Method

#### 2.2.1. Protein Extraction

The oocytes at GV, MI, and MII stages were divided into three groups, with 200 oocytes in each group. The above samples and magnetic beads were added to a centrifuge tube, and 1 mL Lysis Buffer 3, 1 mM PMSF (phenylmethyl sulfonylfluoride), and 2 mM EDTA (EthyleneDiamineTetraaceticAcid) were added. After adding 10 mM DTT (Dithiothreitol) and processing with a tissue grinder and centrifuging at 25,000× *g* for 20 min at 4 °C, the supernatant was removed. Next, 55 mM IAM (iodoacetamide) was added and allowed to stand. Cold acetone was added to precipitate, and this step was repeated until the supernatant was colorless. Finally, Lysis Buffer 3, centrifuged at 25,000× *g* and 4 °C for 20 min to remove supernatant magnetic beads, was added and processed with a tissue grinder, and the supernatant was collected for quantification.

#### 2.2.2. Quality Control of Protein Extraction

The standard protein (0.2 μg/μL BSA) was added to the 96-well microtiter plate at A1 to A10 positions, and different amounts of pure water were added to dilute. Coomassie brilliant blue G-250 quantitative working fluid was added to measure OD595 and create a standard curve. The protein solution to be tested was added to a 96-well microplate, the quantitative working solution was added, and the OD595 was measured. The protein concentration was calculated using a standard curve. Then, 30 μg of protein solution was mixed with loading buffer and heated at 95 °C for 5 min. After centrifugation at 4 °C, 25,000× *g* for 5 min, the supernatant was taken and counted in the spotting wells of 12% SDS polyacrylamide gel. Electrophoresis was performed for 120 min at a constant pressure of 120 V. After electrophoresis, Coomassie brilliant blue staining was performed for 2 h, and then a decolorization solution of 40% ethanol and 10% acetic acid was used 3–5 times for 30 min.

#### 2.2.3. Proteolytic Digestion

Next, 100 μg of protein solution was taken from each sample, and 2.5 μg of trypsin enzyme was added at a ratio of 40:1 protein:enzyme and digested at 37 °C for 4 h. Trypsin was supplemented once, and enzymatic hydrolysis was continued at 37 °C for 8 h. The salt was removed using a Strata X column and the samples were vacuum-dried.

#### 2.2.4. Peptide Labeling

Then, 100 μg of protein solution was taken from each sample, and 2.5 μg of trypsin enzyme was added at a ratio of 40:1 protein:enzyme and digested at 37 °C for 4 h. Trypsin was supplemented once, and enzymatic hydrolysis was continued at 37 °C for 8 h. The salt was removed using a Strata X column and the samples were vacuum-dried.

#### 2.2.5. Peptide Separation

The samples were separated in liquid phase using a Shimadzu LC-20AB liquid-phase system with a 5 μm 4.6 × 250 mm Gemini C18 column. The drained peptide samples were redissolved in 2 mL of mobile-phase A (5% ACN, pH 9.8), injected into the sample, and eluted with a gradient at a flow rate of 1 mL/min, and the elution peaks were monitored at 214 nm, with one fraction collected every minute. The samples were combined with the chromatograms of the eluted peaks to obtain 20 fractions, and then freeze-dried.

#### 2.2.6. High-Performance Liquid Phase

The dried peptide sample was redissolved in mobile-phase A, centrifuged at 4 °C, 20,000× *g* for 10 min to obtain the supernatant, and injected. UltiMate 3000 ultra-high-performance liquid chromatography (UHPLC) was used for separation. The samples were first enriched and desalted using a trap column and then separated using a self-installed C18 column. The separation was performed using a 300 nL min flow rate and effective gradient: 0–5 min, 5% mobile phase B; 5–45 min, mobile phase B increased linearly to 25%; 45–50 min, mobile phase B increased to 35%; 50–52 min, mobile phase B increased to 80%; 52–54 min, 80% mobile phase B; and 54–60 min, 5% mobile phase B. After the separation, the mass spectrometer was connected directly.

#### 2.2.7. Mass Spectrometry Detection

The peptides were separated in the liquid phase and then ionized by a nanoESI source into a Q-Exactive HF X tandem mass spectrometer for DDA mode detection, setting the main parameters as follows: ion source voltage, 2 kV, primary mass spectrometry scan range, 350–1500 m/z, primary resolution, 60,000, secondary mass spectrometry starting m/z, 100; and secondary resolution, 15,000. The HCD fragmentation mode was used to select the top 20 parent ions in terms of intensity from charges 2+ to 5+ for fragmentation. The dynamic exclusion time was set to 30 s, and the AGC was set to 3E6 for the primary and 1E5 for the secondary target.

### 2.3. Bioinformatics Analysis

The data were analyzed by IQuant V2.0.1 (BGl, Shenzhen, China), and the Quant peptide was set to use all unique peptides. Normalization was set to VSN; statistical analysis was set to permutation tests. The obtained raw data were screened for plausible proteins by removing blank values based on the conditions of Score Sequest HT > 0 and uniquepeptide ≥ 1. The differential proteins were screened with a fold change > 1.2 and a Q-value < 0.05. The identified proteome was subjected to GO classification and KOG and KEGG enrichment analyses.

### 2.4. Immunofluorescence Verification

To verify the reliability of the iTRAQ technology, immunofluorescence was used to detect the key differential proteins screened, and two key pathway proteins (SRB1T and HBS1) were selected. Three oocytes were collected at each stage, washed three times, fixed with 2% paraformaldehyde at 25 °C for approximately 1 h, and treated with a fluorescent staining blocking solution containing 0.25% TritonX-100 at 25 °C for 2 h. The oocytes were permeabilized, blocked, and washed three times with DPBS for 5 min each. The primary antibodies THBS1 (ab1823) and SRB1 (ab217318) were added and the oocytes were incubated at room temperature for 2 h, washed with DPBS three times for 5 min, and the corresponding fluorescent secondary antibody was added for incubation at room temperature for 1 h and washed with DPBS three times. Then, 2.5 ng mL^−1^ of DAPI was added, and the oocytes were incubated at room temperature for 3–5 min and washed three times for 5 min. The stained COCs were transferred to the slide, 5 μL of DPBS was added, and the coverslip was sealed. Differentially expressed proteins in oocytes at different stages were detected using a GE DeltaVision Elite live-cell workstation. Each differential protein analysis was repeated three times at different stages of oocyte maturation.

## 3. Results

### 3.1. Mass Spectrometry Identification Results

#### 3.1.1. Basic Identification Information

For the iTRAQ quantification of proteins, three groups of samples were used: GV, MI, and MII oocytes. Three repeated experiments were performed, and 834,458 secondary spectra were generated. A total of 28,204 peptides and 6075 proteins were identified using the ‘1% FDR’ standard. The detailed characteristics of each sample are shown in Table 1.

#### 3.1.2. Protein Relative Molecular Mass Distribution

**Figure 1:**
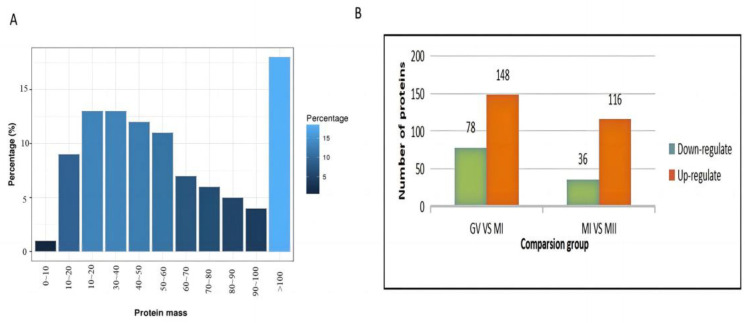
Protein relative molecular mass distribution and differential protein quantity distribution. (**A**) Protein relative molecular mass distribution. (**B**) Differential protein expression quantity distribution.

The relative molecular weights of the identified proteins were mainly concentrated between 10 and 100 kD, as shown in (Figure 1A). This study screened differentially expressed proteins based on protein abundance levels. A total of 226 differentially expressed proteins were identified in GV and MI oocytes, of which 148 were upregulated and 78 were downregulated. In the MI and MII oocytes, 152 differentially expressed proteins were identified, of which 116 were upregulated and 36 were downregulated (Figure 1B).

#### 3.1.3. Peptide Sequence Length Distribution and Protein Abundance Ratio

**Figure 2:**
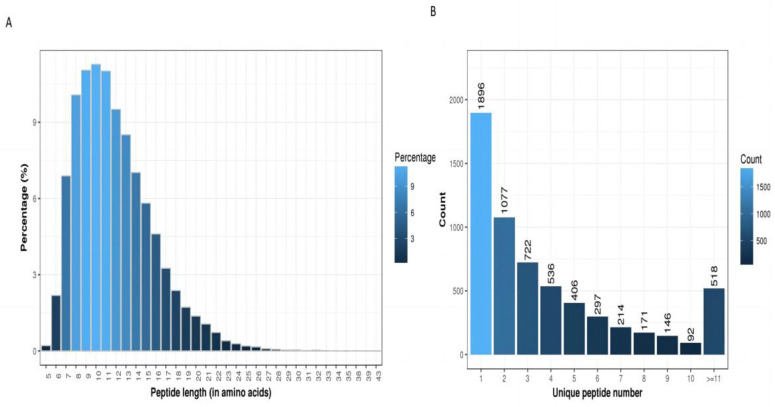
Peptide sequence length distribution. (**A**) Proportion of different peptides. (**B**) Number distribution of specific peptides.

**Figure 3:**
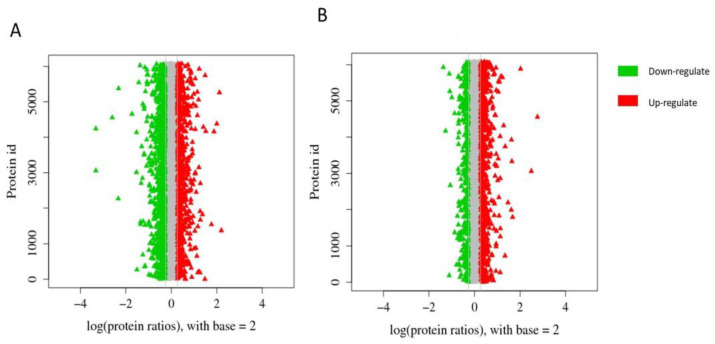
Protein abundance ratio distribution. (**A**) Protein abundance maps of GV and MI oocytes. (**B**) Protein abundance maps of MI and MII oocytes.

When the proportions of peptides of different lengths were counted (Figure 2A), the molecular weight distribution of the peptide molecules was mainly concentrated between 6 and 18. The higher the number of specific peptides, the higher the credibility of the protein. The relationship between the credibility of the protein and the number of its specific peptides (Figure 2B) showed that the reliability of the screened differential proteins was high and that the protein abundance map was based on multiple protein differences. A distribution map was obtained by taking the logarithm of 2 as the base (Figure 3A,B). Green represents the downregulated protein, and red represents the upregulated one.

### 3.2. Bioinformatics Analysis of Differential Proteins

#### 3.2.1. GO Enrichment Analysis of Differential Proteins

**Figure 4:**
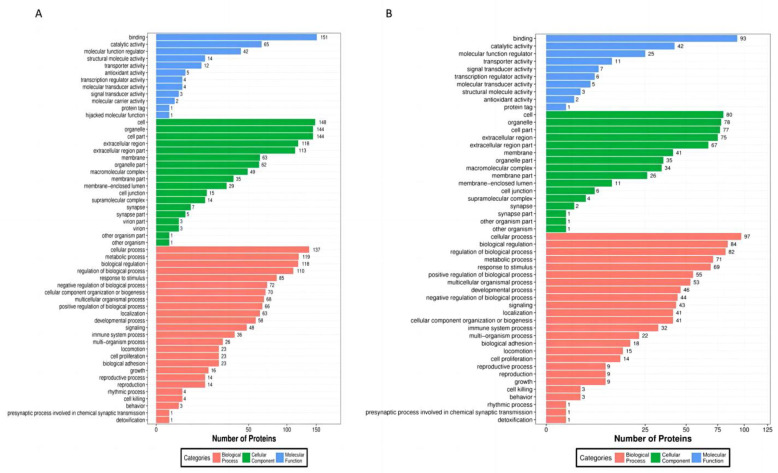
GO enrichment analysis of differential proteins. (**A**) GO enrichment analysis of differential proteins in GV and MI oocytes. (**B**) GO enrichment analysis of differential proteins between MI and MII oocytes.

GO (gene ontology) enrichment analysis of the differentially expressed proteins during the development of oocytes in the GV, MI, and MII phases was performed based on the expression of the differential proteins (Figure 4A,B), which revealed that the molecular functions of the differential proteins were significantly enriched in binding and catalytic activity during the development of oocytes from the GV phase to the MI phase and from the MI phase to the MII phase. Cellular components differed significantly between the organelles and membranes. The biological functions of cellular and metabolic processes differed significantly.

#### 3.2.2. KOG Analysis of Differential Proteins

**Figure 5:**
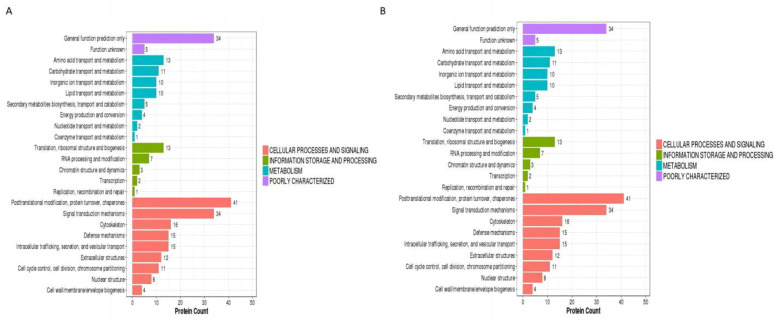
KOG analysis of differential proteins. (**A**) KOG enrichment analysis of differential proteins between GV and MI oocytes. (**B**) KOG enrichment analysis of differential proteins between MI and MII oocytes.

This study compared the identified proteins in the KOG (eukaryotic orthologous groups) database, predicted the possible functions of these proteins, and determined their related functional classifications. The KOG functional annotation divides proteins into four categories: information storage and processing, cellular processes and signal transduction, metabolism, and poorly characterized. KOG functional annotation analysis was performed on the differentially expressed proteins in GV and MI oocytes (Figure 5A). These proteins were primarily involved in amino acid transport and metabolism, carbohydrate transport and metabolism, translation, the post-translational modification of proteins, conversion, and molecular chaperoning. The KOG functional annotation analysis of differentially expressed proteins in MI and MII oocytes (Figure 5B) showed that these proteins were more active in RNA processing and modification, transcription, translation, ribosomal structure and biogenesis, chromatin structure and dynamics, signal transduction mechanisms, post-translational modification of proteins, conversion, and acting as molecular chaperones.

#### 3.2.3. KEGG Analysis of Differential Proteins

**Figure 6:**
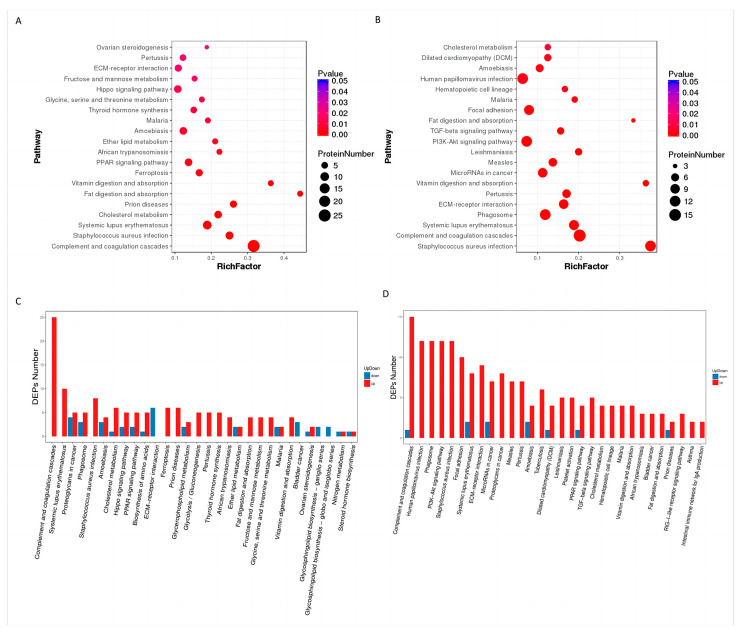
KEGG analysis of differential proteins. (**A**) KEGG enrichment analysis of differential proteins in GV and MI oocytes. (**B**) KEGG enrichment analysis of differential proteins between MI and MII oocytes. (**C**) Analysis of upregulation and downregulation of differential proteins in GV and MI oocytes. (**D**) Analysis of up- and downregulation of differential proteins between MI and MII oocytes.

The KEGG (Kyoto Encyclopedia of Genes and Genomes) enrichment analysis of differentially expressed proteins revealed that the differentially expressed proteins were mainly enriched in specific biochemical metabolic pathways and were involved in signal regulatory pathways. The KEGG analysis of the differentially expressed proteins in GV-and MI oocytes (Figure 6A) showed that they were mainly enriched in the PPAR signaling pathway, Hippo signaling pathway, ECM–receptor interaction, and metabolism-related pathways. To further explore the specific roles of these pathways, the upregulated and downregulated pathways during this period were analyzed (Figure 6C). The phagosome, ferroptosis, Hippo signaling, glycolysis, and metabolic pathways were upregulated, while the ECM–receptor pathway was downregulated. The KEGG analysis of differential proteins between MI and MII oocytes (Figure 6B) showed that they were mainly enriched in immune-related pathways, the PI3K-Akt pathway, the TGF-β pathway, and the phagosome pathway. The upregulated and downregulated protein expression pathways during this period were analyzed (Figure 6D), and the Hippo and PPAR signaling pathways were found to be upregulated. The ECM–receptor pathway was also upregulated.

### 3.3. Immunofluorescence Verification

**Figure 7:**
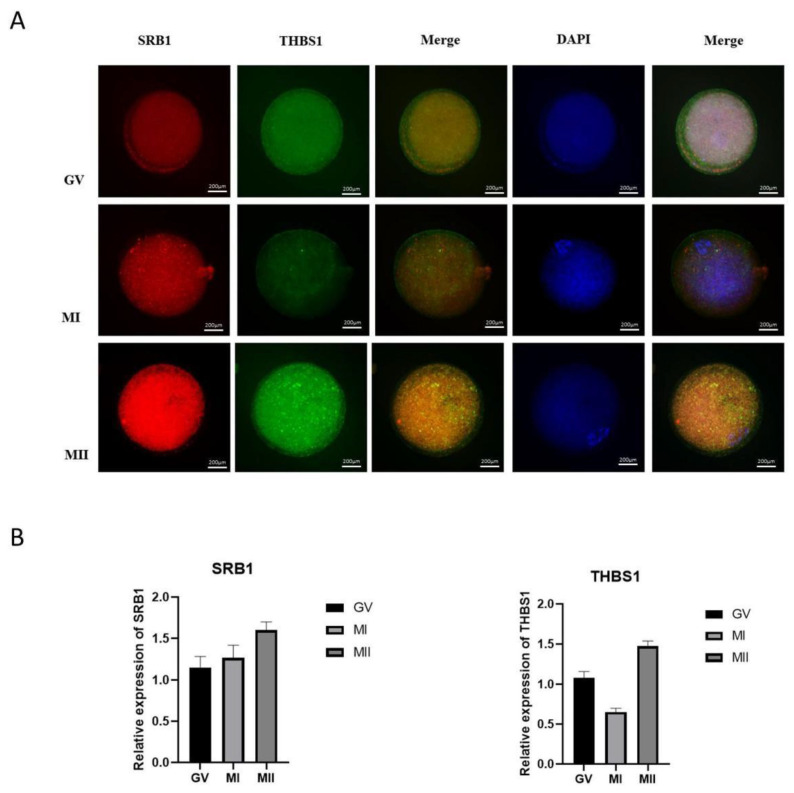
Immunofluorescence verification of differential proteins. (**A**) Immunofluorescence labeling of differential proteins. Scale bar represents 200 µm. (**B**) The expression of differential proteins SRB1 and THBS1 was detected with immunofluorescence assay.

The differential proteins SRB1 and THBS1 were verified with immunofluorescence (Figure 7A). It was found that the differential protein SRB1 was upregulated and THBS1 was downregulated when oocytes developed from the GV stage to the MI stage. When oocytes developed from the MI stage to MII the stage, the differential protein SRB1 was upregulated and THBS1 was upregulated(Figure 7B). These differential proteins were consistent with the results of iTRAQ technology, indicating that the results of this technology were accurate and reliable.

## 4. Discussion

In this study, the differential proteins in the GV, MI, and MII stages of yak oocytes matured in vitro and their biological information were analyzed using iTRAQ technology.

A total of 6075 differentially expressed proteins were screened by comparing the proteomic data of oocytes at the GV, MI, and MII stages. In the GO enrichment analysis of differentially expressed proteins, the ‘binding’ molecular function was the most significant, mainly in terms of protein binding, steroid binding, and lipid binding. This result indicates that the relevant functions in the development of oocytes are completed through molecular binding. These results indicate that the relevant functions in the development of oocytes are completed through molecular binding, which has been confirmed in mice [22], followed by catalytic activity, suggesting that catalytic activity plays an important role in the process of oocyte maturation. Organelle components and extracellular regions account for a large proportion of cells. In the classification of biological processes, the enrichment of metabolic processes was the most significant. This may be because immature oocytes are blocked during the diplotene stage after entering the first meiosis [23]. After reaching sexual maturity, under the stimulation of the LH hormone, the blocked oocytes begin to develop and grow, and the metabolic activities in the oocytes are continuously strengthened to provide a material basis for oocyte maturation and subsequent embryonic development [24,25,26,27]. Oocyte growth requires large amounts of nutrients derived from extracellular cumulus cells. During this process, more proteins are enriched in the extracellular region, whereas oocyte cytoplasmic maturation involves organelle redistribution. In this process, there were more organelle-enriched proteins, especially membrane-like organelles, suggesting that membrane-like organelles such as the endoplasmic reticulum and Golgi apparatus play important roles in oocyte maturation [28,29,30]. After the KOG analysis of differential proteins, some metabolic processes, such as lipid transport and metabolism, amino acid transport and metabolism, and carbohydrate transport and metabolism, were mainly carried out from the GV stage to the MI stage during oocyte maturation. This is because immature oocytes begin to synthesize proteins after hormonal stimulation, such as translation and post-translational modification of proteins that regulate the cell cycle and the accumulation of energy substances by metabolic activities. [31] Differential proteins from MI to MII were enriched in RNA processing and modification, transcription, translation, ribosomal structure and biogenesis, and chromatin structure and dynamics, which may be used to prepare genetic materials for subsequent fertilization and early cleavage [32]. The KEGG analysis of oocytes at each stage showed that from the GV stage to the MI stage, the enriched TOP20 pathways were mainly the PPAR, Hippo, and ECM–receptor interaction pathways. This is because the PPAR signaling pathway is a type of gene expression pattern that transforms nutritional signals into specific genes and controls cellular energy metabolism [33]. In this study, the PPAR signaling pathway was enriched in yak oocytes, which may be related to the regulation of oocyte development by granulosa cells, suggesting that the PPAR signaling pathway plays an important role in maintaining oocyte meiosis [34]. Previous studies have demonstrated that the Hippo pathway plays an important regulatory role in follicular growth and early embryonic development. During follicular growth, GDF9 and BMP15 secreted by oocytes inhibit the Hippo pathway by activating the Smad2 3 signaling pathway in granulosa cells, which enables the continued development of the oocytes. This is because oocytes undergo two physiological arrests during development and maturation. The first arrest occurs during the diplotene stage of the first meiosis, and the second arrest occurs during the second meiosis. The Hippo pathway hinders the formation of microfilament caps in oocytes and destroys their spindle structure, which in turn blocks oocytes; this has been confirmed in mice [35]. In the process of oocyte development from the GV stage to MI, the differentially expressed proteins enriched in metabolic pathways were the most abundant, which was consistent with the results of the GO analysis. The main metabolic pathways were the vitamin, cholesterol, and fat metabolic pathways. Metabolic pathways provide material and energy for the subsequent development of oocytes [36,37]. The phagosome, ferroptosis, and glycolysis pathways are upregulated during oocyte development up to the MI stage. During development, oocytes are surrounded by follicular cells. Through the phagosome pathway, apoptotic cells and cell debris can be removed to prevent the accumulation of harmful substances, maintain the cleanliness of follicles, and provide a healthy microenvironment for oocyte growth [38]. Ferroptosis induces cell death through the excessive accumulation of iron and lipid peroxides. Ferroptosis increases oxidative stress in and around cells during oocyte development, induces lipid peroxidation, and causes follicular atresia. This may be due to limited nutrient availability during oocyte development. Some poorly developed follicles or defective follicular atresia leave sufficient nutrients for the dominant follicles so that oocytes can develop better. In addition, iron ions can regulate the formation and development of oocytes by affecting cell division during oocyte meiosis [39,40]. The glycolytic pathway is upregulated during this process because glucose is an important energy source for oocyte development. Because the oocyte itself has limited use of glucose, it needs to be converted into pyruvate through the glycolytic pathway to provide energy for oocyte development [41].

During the development of oocytes from MI to MII, the TOP20 pathways mainly included the PI3K-Akt, TGF-β, and phagosome pathways. This is because the PI3K-Akt signaling pathway regulates the proliferation, differentiation, and apoptosis of oocytes [42]. It promotes the growth and division of oocytes by upregulating cyclin D1 and cyclin-dependent kinase 4 (CDK4). The activation of the PI3K-Akt signaling pathway promotes the expression of antiapoptotic proteins, such as Bcl-2, to inhibit cell apoptosis. In addition, Akt regulates the localization of the spindle assembly checkpoint protein Mad2, which is essential for the proper arrangement of chromosomes in the equatorial plate during meiosis [43,44]. The TGF-β pathway is involved in cell proliferation, differentiation, tissue development and apoptosis. It plays an important role in oocyte development and maturation. Its mechanism is that type II TGF-β receptor (TβRII) phosphorylates type I TGF-β receptor after binding to the TGF-β ligand activation pathway, activating downstream pathways, such as the Smad pathway, which regulates the development of oocytes. In addition, the TGF-β pathway plays a key role in meiosis. After the first block of oocytes, the TGF-β signaling pathway in the surrounding granulosa cells is activated after LH exposure. This leads to the secretion of downstream factors and the promotion of meiosis [45,46]. Re-enrichment of the phagosome pathway, which is specifically activated after ovulation, promotes follicular rupture and oocyte movement into the fallopian tube. After reaching the oviduct, the phagosome pathway continues to play a role in oocyte transport. The RIG-I-like receptor signaling pathway is upregulated during oocyte development to the MII stage. RIG-I-like receptors (RLRs) belong to a family of intracellular pattern recognition receptors. RIG-I signaling is activated during the oocyte MII stage, leading to the activation of downstream signaling molecules, promotion of the transition from meiosis I to meiosis II, release of the first polar body, and the recovery of meiosis [47].

During oocyte development, the ECM pathway is initially upregulated and then downregulated. Oocyte development is a highly complex process involving the interaction of multiple signaling molecules and biochemical pathways. In the early stages of oocyte development, downregulation of the ECM pathway may occur because, at this stage, cells need to enter the stages of proliferation and differentiation as soon as possible, and the ECM pathway may inhibit these processes. Subsequently, with further oocyte development, the ECM pathway may be upregulated to maintain normal oocyte growth and differentiation. This upregulation may be related to the activation of other signaling molecules and pathways [48,49,50].

## 5. Conclusions

In this study, iTRAQ technology was used to sequence the differential proteomics of the GV, MI, and MII stages of yak oocyte in in vitro maturation, and the dynamic changes in proteomics in this process were analyzed in detail. Protein functions from the GV stage to the MI stage were mainly concentrated in the metabolic pathway, and protein functions from the MI stage to the MII stage were mainly concentrated in the regulation of meiosis and genetic material preparation. This provides basic information for studying protein regulation at each time point during yak oocyte maturation and the molecular mechanisms of in vitro maturation.

## Figures and Tables

**Table 1:** Overview of protein identification results.

Sample Name	Total Spectra	Spectra	Unique Spetra	Peptide	Unique Peptide	Protein
Bos_grunniens	834,458	56,447	50,116	28,204	26,745	6075

## Data Availability

All data presented in this study are available on request from the corresponding authors.
